# Potential Effects of Acidifier and Amylase as Substitutes for Antibiotic on the Growth Performance, Nutrient Digestion and Gut Microbiota in Yellow-Feathered Broilers

**DOI:** 10.3390/ani10101858

**Published:** 2020-10-12

**Authors:** Yibing Wang, Yang Wang, Xiajing Lin, Zhongyong Gou, Qiuli Fan, Jinling Ye, Shouqun Jiang

**Affiliations:** 1 Institute of Animal Science, Guangdong Academy of Agricultural Sciences, State Key Laboratory of Livestock and Poultry Breeding, Key Laboratory of Animal Nutrition and Feed Science in South China, Ministry of Agriculture and Rural Affairs, Guangdong Provincial Key Laboratory of Animal Breeding and Nutrition, Guangzhou 510640, China; wangyibing77@163.com (Y.W.); linxiajing728@sohu.com (X.L.); yozhgo917@163.com (Z.G.); fanqiuli_829@163.com (Q.F.); YeJL2014@163.com (J.Y.); 2 College of Animal Science and Technology, Qingdao Agricultural University, Qingdao 266109, China; yangwang@qau.edu.cn

**Keywords:** acidifier, amylase, growth performance, gut microbiota, yellow-feathered broilers

## Abstract

**Simple Summary:**

Acidifier and amylase were reported to improve the performance of broilers. However, whether acidifier or amylase can be used as substitutes for antibiotics in feed is still unknown. In the present study, benzoic acid (BA), amylase (AL) and their combination (BA+AL) were used to replace the antibiotic (zinc bacitracin, AT). Results showed that the plasma uric acid (UA) was decreased from birds receiving basal diet by all treatments; activity of alkaline phosphatase (AKP) in plasma was lowered by AT, BA, high level AL (AL-H) and BA+AL-H. Plasma activity of lactate dehydrogenase (LDH) was reduced by BA. In the jejunal mucosa, Na^+^K^+^-ATP activity was increased by BA, low level AL (AL-L), AL-H and BA+AL-H. Mucosal activities of total antioxidant capacity (T-AOC) and catalase (CAT) were increased with AL-L and AT supplementation, respectively. Additionally, the relative abundance of *Escherichia coli* (*E. coli*) in the cecal contents was reduced by BA+AL-H and, with the exception of AL-H, all treatments increased the relative abundance of *Lactobacillus*.

**Abstract:**

This study was conducted to evaluate the effects of acidifier (benzoic acid, BA), amylase (AL) and their combination as substitutes for antibiotics on growth performance, antioxidation, nutrient digestion and gut microbiota of yellow-feathered broilers. A total of 1440 twenty-one-day-old broilers were randomly allocated to six treatments. Broilers in the control group (CON) were fed a basal diet, whereas birds in the other five groups were fed the basal diet supplemented with antibiotic (zinc bacitracin, AT, 40 mg/kg), BA (2000 mg/kg), low level AL (AL-L, 300 mg/kg), high level AL (AL-H, 500 mg/kg) and the combination of AL-H and BA (BA+AL-H). The experimental animals were killed at the end of the trial (21 day-63 day) then blood samples were collected from two birds per pen. Bird weight, feed intake and survival rate were recorded on pen basis. Growth performance was not significantly influenced by AT, BA, AL-L, AL-H or BA+AL-H. Plasma uric acid (UA) was decreased from CON by all treatments; the activity of AKP in plasma was also lowered by AT, BA, AL-H and BA+AL-H. Plasma activity of LDH was reduced by BA. In the jejunal mucosa, Na^+^K^+^-ATP activity was increased by BA, AL-L, AL-H and BA+AL-H. Mucosal activities of T-AOC and CAT were increased with AL-L and AT supplementation, respectively. Additionally, the relative abundance of *Escherichia coli* (*E. coli*) in the cecal contents was reduced by BA+AL-H and, with the exception of AL-H, all treatments increased the relative abundance of *Lactobacillus*. In conclusion, dietary AT, BA, AL-L, AL-H or BA+AL were effective in improving the antioxidant capacity, nutrient digestion and gut microbiota composition. No significant differences were observed in the tested variables between AT and other treatments, indicating that BA, AL and their combination may be alternatives to dietary inclusion of zinc bacitracin. Dietary addition of 500 mg/kg AL and 2000 mg/kg BA was an optimum supplementation dose.

## 1. Introduction

Dietary antibiotics are known to promote growth performance and control gastrointestinal infections in animals [1,2]. The ban on use of antibiotics in feed has largely resulted from the emergence of resistant bacteria and the potential for drug residues being present in animal products [3]. Thus, the search for alternatives to antibiotics that can improve performance and nutrient utilization is an urgent contemporary need.

Acidifiers, including organic acids such as formic, acetic, propionic, butyric, fumaric, citric and benzoic [4], lower the pH of feed and can protect against microbial contamination and spoilage [5,6]. They can also increase performance [7,8], improve structure of the gut microbiota [8,9,10], promote indices of nutrient digestibility [4,8] and regulate immune function [4] of broilers. Benzoic acid (BA) is superior to other acids as it has the beneficial effect of reducing coliforms in both the stomach and small intestine, as well as reducing *Salmonella* Typhimurium [11,12,13].

Although chicks are adapted to starch-based diets soon after hatching, the high feed intake of modern broilers may produce a physiological limitation to starch digestion [14,15]. Thus, amylase (AL) supplementation in corn (maize)-based diets improved the growth performance, nutrient digestibility [16] and energy utilization of broilers [15].

The broiler performance can also be affected by the activities of endogenous intestinal enzymes. Brush border enzymes, such as alkaline phosphatase (AKP), creatine kinase (CK) and dehydrogenase (LDH), are commonly served as markers of intestinal damage [17]. Besides, pepsin, trypsin and chymotrypsin are enzymes that affects protein and amino acids digestibility [18,19]. Thus, these parameters can reflect the intestinal health and nutrient digestibility of broilers.

Despite of these findings, to our knowledge, no research has been done to compare the roles of acidifiers or AL and examine their synergistic effect in the health of broilers. The current study was conducted, therefore, to directly investigate the dietary use of BA and AL, separately or in combination, on growth performance, nutrient utilization and gut microbiota of yellow-feathered broilers. These birds are widely used in South Asia where the meat characteristics are favored by local consumers; they represent almost half of the meat chickens marketed in China.

## 2. Materials and Methods

### 2.1. Chicken Husbandry and Diets

The experimental protocol was approved by the Animal Care Committee of the Institute of Animal Science, Guangdong Academy of Agriculture Science, Guangzhou, P. R. China, with the approval number of GAASISA-2014023. A total of 1440 twenty-one-day-old yellow-feathered male broilers (Lingnan, an improved meat-type breed, obtained from Guangdong Academy of Agriculture Science, Guangzhou, China) with similar hatchling weights were randomly allocated to 6 treatments, each with 6 replicates of 40 birds per pen. The composition and calculated nutrient content of basal diets in the first 3 weeks of raising is presented in Table S1. The controls (CON) received the basal diets, composition and nutrient contents of which are provided in Table 1. These diets satisfy the recommendations of Chinese Feeding Standard of Chicken (Ministry of Agriculture, PRC, 2004) for grower (day 21 to 42) and finisher (day 43 to 63) phase yellow-feathered broilers. The other 5 treatments were: AT (40 mg/kg zinc bacitracin); BA (2000 mg/kg benzoic acid); AL-L (300 mg/kg amylase); AL-H (500 mg/kg amylase); and BA+AL-H (2000 mg/kg benzoic acid + 500 mg/kg amylase). Benzoic acid and amylase were purchased from Zhejiang Academy of Agricultural Sciences. Fresh water and feed were provided ad libitum until d 63, the typical marketing age for this strain. Daylight was eliminated and replaced with 18-h lighting from incandescent bulbs. The temperature of the room was maintained at 26 °C.

### 2.2. Measurement of Growth Performance

The amounts of provided and refused feed were measured daily on a replicate basis to calculate the average daily feed intake (ADFI), including adjustments for any dead birds. Mortality of birds was recorded daily. Final body weight (BW) were measured at d 42 and 63 to calculate average daily BW gain (ADG), and feed conversion rate (FCR) on a per replicate basis.

### 2.3. Sample Collection

On d 63, after 12 h of feed-withdrawal, the birds were electrically stunned and exsanguinated (DMJ, NingguangMachinery Co., Ltd., Nanjing, China). Blood samples were collected in 5 mL heparinized tubes from the jugular vein of 2 birds per replicate. Plasma was obtained by centrifugation at 1000× *g* for 15 min at 4 °C. Mid-jejunal segments were carefully dissected, rinsed with sterilized saline opened lengthwise. Then the mucosa was collected by gentle scraping and snap-frozen in liquid N_2_, homogenized with ice-cold physiologic saline (1:10, *v*/*v*) and centrifuged at 2000× *g* for 10 min. Cecal digesta was obtained by massaging the tract. All samples were placed immediately in liquid nitrogen and then held at −80 °C.

### 2.4. Analysis of Biochemical Indexes

The plasma variables: uric acid (UA), AKP, inducible nitric oxide synthase (iNOS), CK, LDH, D-xylose, and the jejunal variables: pepsin, trypsin, chymotrypsin, amylase, Na^+^K^+^-ATPase, total antioxidant capacity (T-AOC) and catalase (CAT) were determined spectrophotometrically using commercial kits (Nanjing Jiancheng Institute of Bioengineering, Nanjing, China).

### 2.5. Gut Microbial Enumeration

The quantitative real-time PCR of *Lactobacillus*, *E. coli*, *Enterococcus* and total bacteria was achieved by using a qPCR assay. Genomic DNA were extracted from cecal contents using TIANamp Stool DNA Kits (Tiangen Biotech, Beijing, China). The primers used are shown in Table S2. Real-time PCR was performed using SYBR Premix-Ex Taq II (TAKARA) and the ABI 7500 real-time PCR system (Applied Biosystems, Carlsbad, CA, USA) [20]. Serial dilutions of linearized plasmid DNA (pMD™19-T Vector Cloning Kit, Takara, JAPAN) inserted with respective bacterial amplicons were used to construct a standard curve. The concentrations of plasmid DNA were measured by NanoDrop1000 (Thermo Fisher Scientic Inc. Waltham, MA, USA) before the serial dilutions. The number of target DNA copies was calculated from the mass of DNA. Bacteria numbers were expressed as log10 (genomic DNA copy number)/g ceca.

### 2.6. Statistical Analysis

Effects of treatment were examined by one-way analysis of variance (ANOVA) in SPSS 20.0 for Windows; all percentage data were arcsine transformed before analysis. When treatment effects were significant (*p* < 0.05), Tukey’s multiple range tests were used to compare pairs of means. Tabulated results are shown as means with SEM derived from the ANOVA error mean square.

## 3. Results

### 3.1. Growth Performance

No significant effects of treatment were observed for growth performance variables (Table 2). These consisted of initial weight at d 21, BW at d 42 and 63, and ADG, ADFI, FCR and survival rates for the three periods.

The results shown in Table 3 indicated that plasma UA content was decreased significantly (*p* < 0.01) from CON by all treatments. The plasma activity of AKP was also lowered significantly (*p* < 0.05) by AT, BA, AL-H and BA+AL-H while activity of LDH was only reduced significantly (*p* < 0.05) from CON by BA. There were no effects of treatment on plasma activities of iNOS, CK and the concentration of D-xylose.

### 3.2. Biochemical Indices in Jejunal Mucosa

Activities of selected digestive enzymes were examined in jejunal mucosa (Table 4). There were no effects of treatment in the activities of pepsin, trypsin, chymotrypsin and amylase but activity of Na^+^K^+^-ATPase was significantly increased (*p* < 0.05) by BA, AL-L, AL-H and BA+AL-H. There were significantly increased activities of T-AOC with AL-L and CAT with AT compared to the control (*p* < 0.05).

### 3.3. Gut Microbial Enumeration

Dietary supplementation with antibiotic (AT), acidifier (BA) and/or amylase (AL-L, AL-H) did not change the total number of total bacteria in cecal contents significantly. Nevertheless, the count of *E. coli* was significantly reduced by BA+AL-H significantly (*p* < 0.05). Moreover, except for AL-H, all treatments increased (*p* < 0.05) the count of *Lactobacillus* significantly. The count of *Enterococcus* was not affected significantly by treatment (*p* > 0.05) (Table 5).

## 4. Discussion

Dietary supplementation with acidifiers improves the growth performance of broilers. For example, the addition of acetic, citric, and lactic acid increased BWG and decreased FCR [21]. Acidifiers, consisting of formic, propionic and acetic acids, improved BWG and FCR during the finisher period [4]. However, differently, Gunal et al. found no significant improvement in growth performance from dietary acidification [22]. Giannenas et al. also reported that the growth performance was not affected by BA supplementation [23]. The same was found in the present study. Amylase (AL), in combination with other digestive enzymes, such as xylanase and protease has been investigated in broilers. The combination of xylanase, AL and protease (XAP) increased ADG by 12% and reduced FCR by 0.09 units [24]. Amerah et al. also found a synergistic effect between xylanase, AL and protease on broiler performance, whereas AL alone had no significant effects on performance [25]. Stefanello et al. also found that AL did not affect BWG and FCR significantly during1 to 21 d, 22 to 40 d and 1 to 40 d [15]. The present results, using yellow-feathered broilers, showed no significant effect on performance with the AL-H and AL-L treatments, nor any synergistic effect between BA and AL-H on the ADG, ADFI and FCR. According to Svihus, the specific diet properties, cereal type, inclusion level and bird related factors might contribute to the variation in the outcomes of supplementing poultry diets with amylase [26].

Uric acid (UA) is a potent antioxidant [27] and, in the present study, the plasma UA concentration was decreased by all treatments, compared to CON. The decrease in UA was not considered to be pathological, because no symptoms, such as impaired growth performance, villous congestion and hemorrhage were observed. Many studies [28,29] have found the feed additives, such as probiotics, also decreased non-protein nitrogen in chicken blood, including uric acid, ammonia, and urea. Plasma AKP activity in broilers was higher after artificial infection [30] and previous work from this laboratory demonstrated that supplementation with protocatechuic acid lowered blood AKP, indicating less inflammation [31]. Similarly, the present reduction in plasma AKP activity by AT, BA, AL-H and BA+AL-H, implies decreased inflammation. LDH leakage is related to the damage of tight junction integrity [32,33] and decreased plasma LDH activity in BA-treated broilers in the present study suggested improvement of the intestinal barrier.

The activity of digestive enzymes is an important factor affecting growth performance [34] yet the current results showed no influence of adding AT, BA, AL-L, AL-H or BA+AL-H on the jejunal mucosal activities of pepsin, trypsin, chymotrypsin or amylase. Similarly, the jejunal trypsin, amylase or lipase were not altered significantly by acidifier [4]. Unfortunately, no other studies using acidifiers have reported data on digestive enzyme activities in broilers to serve for comparison with our results. As for the addition of AL, Jiang et al. found that 750 and 2250 mg/kg AL significantly increased the activities of intestinal AL, protease and trpsin [35]. Ma et al. observed that dietary amylase (3000 and 6000 U/kg) could significantly increase AL activity compared with non-amylase diets [34]. Therefore, as far as we are concerned, the data on the endogenous digestive enzymes may differ from the dosage and activity of exogenous AL used. The pump Na^+^K^+^-ATPase is involved in the digestive and absorptive process in poultry [36]. Except for the AT treatment, there was higher jejunal mucosal activity of Na^+^K^+^-ATPase with BA, AL-L, AL-H and BA+AL-H treatments, consistent with increased capacity for nutrient absorption. Although the aforementioned result showed plasma UA, an antioxidant indicator, reduced by all treatments, increased jejunal T-AOC with AL-L, and jejunal CAT activity with AT group suggested that these treatments enhanced jejunal antioxidant capacity in broilers.

Organic acids have a great impact on the function of the gastrointestinal tract of poultry by changing the microbial population via depolarization of the bacterial membrane and increasing internal acidity of the bacteria [37]. The gut microbiota can also be affected by dietary composition and its digestibility [38]. Because AL appeared to improve digestive capacity, it was hypothesized that dietary supplementation with acidifier and AL may alter the population structure of gut microbiota. In the current study, the total bacterial number was unchanged by treatment but the BA+AL-H treatment had much reduced *E.coli* count, while AT, BA, AL-L and BA+AL-H treatments increased the presence of *Lactobacillus*. Different results were obtained by Heidari et al., where counts of *E. coli* and *Lactobacillus* in broilers were not influenced by acidifier [39]. Torrallardona et al. showed that BA improved performance of piglets, and this was associated with a better structure of the gut microbiota structure [40]. Administration of amylase or amylase combined with amylopectase and glucoamylase to broilers enhanced the count of cecal *Lactobacillus* [37]. It is reported that the enhanced populations of *Lactobacilli* may be competed with the pathogens such as *E. coli* for attachment sites on the gut surface [41]. Thus, these results suggest that BA and AL supplementation altered the gut microbiota composition in a way that benefits the host.

## 5. Conclusions

Although the experimental treatments did not affect the growth performance of broiler chickens, the plasmal variables, jejunal indices and gut microbiota were improved by AT, BA, AL-L, AL-H and BA+AL-H. No significant differences were observed between birds receiving in-feed AT and other additive treatments. Dietary supplementation with BA, AL and their combination, therefore, had similar effects on the health of broilers as zinc bacitracin. However, whether BA, AL and their combination can be used as alternatives to antibiotics needs further study.

## Figures and Tables

**Table 1:** Composition and calculated nutrient content of basal diets (as-fed basis).

Items	Grower (d 22 to 42)	Finisher (d 43 to 63)
Ingredients, %		
Corn	64.20	67.20
Soybean meal	23.40	18.50
Feather meal	3.30	3.50
Soybean oil	3.00	4.40
L-Lys.HCL (78%)	0.14	0.14
DL-Met (99%)	0.09	0.07
Limestone	1.17	1.10
CaHPO_4_·2H_2_O	1.65	1.40
NaCl	0.30	0.30
Zeolite powder	1.75	2.39
premix ^1^	1.00	1.00
Total	100.00	100.00
Nutrient contents ^2^		
ME, MJ/kg	12.54	12.96
CP, %	19.00	17.00
Lysine, %	0.98	0.85
Methionine, %	0.36	0.31
Met+Cys %	0.73	0.65
Thr, %	0.75	0.67
Trp, %	0.21	0.18
Ile, %	0.77	0.69
Ca, %	0.90	0.80
Non-phytate phosphorus, %	0.40	0.35

^1^ Premix provided the following per kilogram of diets during 22 to 42 d of age: VA 15,000 IU, VD_3_ 3300 IU, VE 20 IU, VK_3_ 6 mg, VB_1_ 1.8 mg, VB_2_ 9 mg, VB_6_ 3.5 mg, VB_12_ 0.01 mg, chloride 500 mg, niacin 60 mg, pantothenic acid 16 mg, folic acid 0.55 mg, biotin 0.15 mg, Fe 80 mg, Cu 8 mg, Mn 80 mg, Zn 60 mg, I 0.35 mg and Se 0.3 mg. Premix provided the following per kilogram of diets during 43 to 63 d of age: VA 10,000 IU, VD_3_ 1,000 IU, VE 20 IU, VK_3_ 6.0 mg, VB_1_ 3.0 mg, VB_2_ 9.0 mg, VB_6_ 6.0 mg, VB_12_ 0.03 mg, chloride 1,000 mg, niacin 60 mg, pantothenic acid 18 mg, folic acid 0.75 mg, biotin 0.10 mg, Fe 80 mg, Cu 12 mg, Mn 100 mg, Zn 75 mg, I 0.35 mg and Se 0.15 mg. ^2^ Values were calculated from data provided by Feed Database in China (2012).

**Table 2:** Effects of acidifier and amylase on growth performance of yellow-feathered broilers ^1^.

Variables	CON	AT	BA	AL-L	AL-H	BA+AL-H	SEM	*p* Value
21 to 42 d								
Initial weight, g	594.67	594.44	593.89	595.00	594.44	595.00	0.88	0.943
Final weight, g	1459.33	1456.67	1399.33	1442.67	1444.93	1411.24	19.53	0.225
ADG, g	43.23	43.11	40.27	42.37	42.48	40.80	0.97	0.224
ADFI, g	109.80	108.68	104.97	108.70	108.15	107.69	1.45	0.385
FCR	2.49	2.52	2.62	2.62	2.59	2.61	0.05	0.619
Survival rate, %	100.00	100.00	100.00	100.00	100.00	98.89	0.45	0.435
43 to 63 d								
Final weight, g	2201.60	2195.09	2122.44	2186.70	2201.44	2170.57	44.83	0.837
ADG, g	41.24	41.02	40.66	42.63	43.03	45.13	2.21	0.751
ADFI, g	134.87	134.25	132.24	133.09	137.84	133.52	3.13	0.876
FCR	3.21	3.27	3.26	3.13	3.25	3.04	0.11	0.561
Survival rate, %	98.46	97.44	100.00	98.72	98.72	100.00	1.10	0.825
21 to 63								
ADG, g	42.24	42.07	40.91	43.06	43.42	44.00	1.40	0.785
ADFI, g	122.33	121.47	119.64	122.09	124.37	121.68	1.99	0.811
FCR	2.90	2.89	2.92	2.84	2.88	2.79	0.06	0.688
Survival rate, %	98.46	97.44	100.00	98.72	98.72	98.89	1.19	0.631

^1^ Values are means of 6 replicates per treatment, each with 40 broilers. CON = the control group, AT = the antibiotic (zinc bacitracin), BA = benzoic acid, AL-H = high level amylase, AL-H = high level amylase, AL-L = low level amylase. SEM = standard error.3.2. Biochemical Indices in Plasma.

**Table 3:** Effects of acidifier and amylase on plasma biochemical indexes of yellow-feathered broilers ^1^.

Items	CON	AT	BA	AL-L	AL-H	BA+AL-H	SEM	*p* Value
UA, umol/L	301.13 ^a^	209.42 ^b^	182.00 ^b^	217.30 ^b^	216.82 ^b^	170.94 ^b^	26.78	0.006
AKP, U/100mL	32.47 ^a^	15.10 ^b^	12.70 ^b^	22.06 ^ab^	14.41 ^b^	16.00 ^b^	5.41	0.036
iNOS, U/mL	2.97	2.92	2.92	3.04	3.12	2.92	0.30	0.998
CK, U/mL	2.02	1.97	1.58	2.13	2.03	1.78	0.24	0.621
LDH, U/mL	3.55 ^a^	3.25 ^ab^	3.05 ^b^	3.52ab	3.50 ^ab^	3.42 ^ab^	0.22	0.024
D-xylose, mmol/L	0.47	0.52	0.47	0.49	0.58	0.44	0.05	0.280

^1^ Values are means of 12 samples with 2 birds per replicate. ^ab^ Mean value within a role with no common superscript differ significantly (*p* < 0.05). CON = the control group, AT = the antibiotic (zinc bacitracin), BA = benzoic acid, AL-H = high level amylase, AL-H = high level amylase, AL-L = low level amylase. UA = uric acid, AKP = alkaline phosphatase, iNOS = inducible nitric oxide synthase, CK = creatine kinase, LDH = lactate dehydrogenase, SEM = standard error.

**Table 4:** Effects of acidifier and amylase on jejunal biochemical indexes of yellow-feathered broilers ^1^.

Variables ^2^, U/mg prot	CON	AT	BA	AL-L	AL-H	BA+AL-H	SEM	*p* Value
Pepsin	1.05	0.665	1.12	0.93	1.20	1.25	0.23	0.594
Trypsin	64.90	79.95	83.44	80.29	63.81	80.56	10.91	0.597
Chymotrypsin	1.16	0.72	1.16	0.79	0.99	1.09	0.15	0.499
Amylase	0.77	0.63	0.68	0.65	0.57	0.58	0.19	0.950
Na^+^K^+^-ATPase	1.11 ^b^	1.20 ^ab^	1.62 ^a^	1.63 ^a^	1.57 ^a^	1.87 ^a^	0.11	0.031
T-AOC	1.40 ^ab^	1.42 ^ab^	1.20 ^b^	1.583 ^a^	1.39 ^ab^	1.185 ^b^	0.19	0.015
CAT	1.35 ^b^	2.86 ^a^	2.20 ^ab^	1.99 ^ab^	1.66 ^ab^	2.12 ^ab^	0.50	0.016

^1^ Values are means of 12 birds with 2 birds per replicate. ^2^ Units/mg prot. ^ab^ Mean value within a role with no common superscript differ significantly (*p* < 0.05). CON = the control group, AT = the antibiotic (zinc bacitracin), BA = benzoic acid, AL-H = high level amylase, AL-H = high level amylase, AL-L = low level amylase. T-AOC = total antioxidant capacity, CAT = catalase, SEM = standard error.

**Table 5:** Effects of acidifier and amylase on the relative abundance of cecal bacteria of yellow-feathered broilers ^1^.

Variables ^2^	CON	AT	BA	AL-L	AL-H	BA+AL-H	SEM	*p* Value
Total bacteria	12.85	12.85	13.15	13.11	13.01	13.11	0.22	0.544
*E. coli*	10.29 ^a^	9.67 ^ab^	9.33 ^ab^	9.66 ^ab^	9.84 ^ab^	8.93 ^b^	0.46	0.041
*Enterococcus*	7.94 ^ab^	7.59 ^ab^	7.75 ^ab^	8.37 ^a^	7.44 ^b^	7.80 ^ab^	0.34	0.026
*Lactobacillus*	11.18 ^b^	11.69 ^a^	11.69 ^a^	11.80 ^a^	11.61 ^ab^	11.71 ^a^	0.27	0.008

^1^ Values are means of 12 birds with 2 birds per replicate. ^2^ Data are log10(CFU)/g. ^ab^ Mean value within a role with no common superscript differ significantly (*p* < 0.05). CON = the control group, AT = the antibiotic (zinc bacitracin), BA = benzoic acid, AL-H = high level amylase, AL-H = high level amylase, AL-L = low level amylase. SEM = standard error; *E. coli = Escherichia coli.*

## Supplementary Materials

The following are available online at https://www.mdpi.com/2076-2615/10/10/1858/s1, Table S1: Composition and calculated nutrient content of basal diets (as-fed basis) in first 3 weeks of raising; Table S2: Sequence of primers used for the qPCR analysis.
