# Supplementary material for: Potential Effects of Acidifier and Amylase as Substitutes for Antibiotic on the Growth Performance, Nutrient Digestion and Gut Microbiota in Yellow-Feathered Broilers
Source: Animals (Basel). 2020 Oct 12;10(10):1858. doi: 10.3390/ani10101858 (PMC7600580; doi:10.3390/ani10101858)
Supplement: Supplementary file 1 [file animals-10-01858-s001.pdf]

Supplementary Materials:

Table S1. Composition and calculated nutrient content of basal diets (as-fed basis) in first 3 weeks of raising.

**Items**	**Starter (d 1 to 21)**
Ingredients, %	
Corn	60.40
Soybean meal	25.65
Feather meal	4.80
Soybean oil	2.00
L-Lys.HCL (78%)	0.16
DL-Met (99%)	0.18
Limestone	1.20
$CaHPO_4 \cdot 2H_2O$	1.90
NaCl	0.30
Zeolite powder	2.41
premix[1]	1.00
Total	100.00
Nutrient contents[2]	
ME, MJ/kg	12.12
CP, %	21.00
Lysine, %	1.16
Methionine, %	0.42
Met+Cys %	0.84
Thr, %	0.78
Trp, %	0.19
Ile, %	0.78
Ca, %	1.00
Non-phytate phosphorus, %	0.46

[1] Premix provided the following per kilogram of diets during 1 to 21 d of age: VA 15,000 IU, VD_3 3,300 IU, VE 20 IU, VK_3 6 mg, VB_1 1.8 mg, VB_2 9 mg, VB_6 3.5 mg, VB_{12} 0.01 mg, chloride 500 mg, niacin 60 mg, pantothenic acid 16 mg, folic acid 0.55 mg, biotin 0.15 mg, Fe 80 mg, Cu 8 mg, Mn 80 mg, Zn 60 mg, I 0.35 mg, and Se 0.3 mg.[2] Values were calculated from data provided by Feed Database in China (2012)

Table S2. Sequence of primers used for the qPCR analysis

Items	Primer sequence (5′-3′)	Annealing tempreture (°C)
Total bacteria	F: CGGCAACGACGCAACCC<br>R: CCATTGTAGCACGTGTGTAGCC	60
E.coli	F: GTTAATACCTTTGCTCATTGA<br>R: ACCAGGGTATCTAATCCTGTT	50
Enterococcus	F: CGATGAGTGCTAGGTGTTGGA<br>R: CAAGATGTCAAGACCTGGTAAG	60
Lactobacillus	F: CATGCCGCGTGTATGAAGAA<br>R: CGGGTAACGTCAATGAGCAAA	60
